# Design of Nano-Structured Micro-Thermoelectric Generator: Load Resistance and Inflections in the Efficiency

**DOI:** 10.3390/e21030224

**Published:** 2019-02-27

**Authors:** Carlos Alberto Badillo-Ruiz, Miguel Angel Olivares-Robles, Jose Jorge Chanona-Perez

**Affiliations:** 1 Instituto Politecnico Nacional, Seccion de Estudios de Posgrado e Investigacion, Escuela Nacional de Ciencias Biologicas, Ciudad de Mexico 11340, Mexico; 2 Instituto Politecnico Nacional, Seccion de Estudios de Posgrado e Investigacion, Escuela Superior de Ingenieria Mecanica y Electrica Unidad Culhuacan, Coyoacan, Ciudad de Mexico 04430, Mexico

**Keywords:** thermoelectric, micro-generator, Thomson effect, nanostructuring

## Abstract

In recent years the interest for the harvest of energy with micro thermoelectric generators (μTEG) has increased, due to its advantages compared to technologies that use fossil fuels. There are three ways to improve the performance of the device, by modifying its structure, type of material and operation control. In this study, the role of the load resistance $R_{L}$ on the performance of a μTEG with nanostructured materials is investigated. The interaction of the load resistance with the thermoelements exhibits interesting features, arising from the coupling of the temperature-dependent electrical and thermal transport properties at different temperature ranges and the architecture of nanostructured thermoelectric materials. This coupling results in inflections on the efficiency, i.e., maximum and minimum values of the efficiency at higher temperatures, 600–900 K. We show the explicit dependence of the performance of the μTEG in terms of the load resistance and discuss the underlying physics. The unusual features of the efficiency of nanostructured thermoelectric materials are a result of the behavior of the power factor and the nonequilibrium properties of the system. We also analyze the effect of the geometric shape of the thermoelements on the device. We determine the performance of the μTEG, evaluating the generation power and its efficiency. The results show that the efficiency of the device can decrease or increase depending on the value of $R_{L}$, while the power decreases with an increase of the load resistance.

## 1. Introduction

The thermoelectric generating devices are solid-state devices with significant advantages, since it is an energy harvester without either moving parts or the need for continuous maintenance, making it a sustainable and environmentally friendly alternative. However, the efficiency of the thermoelectric generators (TEG) is around 5 percent [1].

Many methods to increase the efficiency of thermoelectric devices focuses on the manipulation of materials to optimize the figure of merit (ZT). Therefore, in recent years a strategy has been developed to increase its performance. The approach is based on optimizing the materials by improving the power factor, that is, increasing the Seebeck coefficient without overshadowing the electrical conductivity. This can be done with the modification of the structure of the band by degeneration of many valleys, electronic resonance states and bipolar effect, among others. Most of the high-performance materials are nanostructured and have an impact of reducing the thermal conductivity of the lattice. This allows for achieving figures of merit greater than one with temperatures of up to 800 K [2,3,4].

Another way to improve efficiency is based on controlling the operating conditions of the TEG, establishing a temperature difference between the TEG reservoirs, submitting to heat transfer by convection or modifying the load resistance, among others. Recent research has shown that the power of the thermoelectric generators is affected by the load resistance. Experimental data show that as the load resistance increases the power of the generator decreases [5,6,7,8,9,10].

Recently, it has been shown that performance parameters such as efficiency and output power depend on the relationship between the load resistance and the internal resistance of the TEG defined as $m=R_{L}/R_{int}$. Lamba et al. [11] showed the efficiency and power of the TE depending on load resistance and length of thermoelectric. They determined that the optimal relationship between resistances is between m = 1–3 because you can get maximum efficiency and maximum power of the thermoelectric in this range. They also showed that the performance of the TEG is affected by its geometric factor, obtaining a better performance with the increase in length.

Recent research has shown that a geometrical structure with slightly inclined thermoelements can provide a better performance with respect to traditional thermoelements, that is, it shows a slight improvement in the efficiency and power of the thermoelectric module, because this shape can suppress the heat transfer from the hot side to the colder side [12,13].

The maximum efficiency of a thermoelectric material is given by $ZT=\alpha^{2}\sigma T/\kappa$, as a function of its thermoelectric properties, with coefficient Seebeck (α(T)), thermal conductivity (κ(T)) and electrical conductivity (σ(T)) being dependent on temperature. The design of nanostructured materials is a strategy that has shown attractive results for its application, the decrease in thermal conductivity and a figure of merit for above the threshold value of two with high temperatures [14,15].

Other materials have been studied through the nanostructuring technique, for example, $\beta -Zn_{4}Sb_{3}$. The thermoelectric properties, Seebeck coefficient, thermal and electrical conductivity for different carrier concentrations of $\beta -Zn_{4}Sb_{3}$ material are highly attractive due to its low cost and toxicity as an alternative to tellurium-based materials. However, it has been reported that this material exhibits figures of merit below ZT=1 in a maximum temperature range of 500 K [16]. Also, defects in the material contribute to the diffuse dispersion of phonons, and defects such as dislocations can reduce thermal conductivity but impair electrical conductivity. Thus, point defects significantly affect the properties of the $\beta -Zn_{4}Sb_{3}$ material which, depending on the concentration of Zn, gives rise to nanovoids and nanograins originating at different phases of the material at micrometer scale and nanometer scale [17].

The potential of techniques such as nanostructuring has benefited from several approaches, such as band engineering and defect optimization. The improvement of efficiency in materials is based on the reduction of the characteristic length of the nanostructure, which relies on the belief that phonons mean free paths (MFPs) are typically much longer than electrons. A high MFP value is necessary since it implies high mobility of electrons, which leads to a better performance. In the nano-structuring, the introduction of irregularities in the network by alloy plays a vital role, because this causes a sufficient disorder that produces the dispersion of the phonons at high temperatures [18].

Bulk nanostructures allow the effective scattering of phonons, however, phonons with long free mean trajectories are not affected. In a recent work Biswas et al. [19] showed that by controlling and fine-tuning the mesoscale architecture of p-type PbTe material endotaxially nanostructured with SrTe at a concentration of 4 mole percent and mesostructured with powder processing, phonons that carry heat with long paths could undergo scattering. Therefore, when considering the different hierarchies of length scales and the different sources of dispersion of them, the maximum reduction in thermal conductivity is achieved. Biswas combines three main effects, (a) effects of alloy doping at the atomic scale, (b) endotaxial nanostructure and (c) mesoscale grain limit control, resulting in the maximum dispersion of phonons at high temperatures compared to a nanostructure without doping. Also, an increase in ZT beyond the threshold of 2 highlights the role of, and need for, multiscale hierarchical architecture in controlling phonon scattering in bulk thermoelectrics, and offers a realistic prospect of the recovery of a significant portion of waste heat [19].

Therefore, in this work the materials proposed by Biswas et al. [19] are studied since they demonstrate interesting characteristics for energy harvesting applications, the influence of the geometrical form of the thermoelements as well as the role of the load resistance on the performance of μTEG is also studied.

The present work is organized as follows. In Section 2, we present our model of a μTEG where the equations that govern the system and the thermoelectric properties of each material are described in detail. Our results and discussion about the effect of the shape factor and load resistance on efficiency and power are presented in Section 3. The conclusions obtained from the study are explained in Section 4.

## 2. Methods

In the framework of Onsager’s linear theory, the interaction of heat and flow of electric current in a thermoelectric process is described regarding the kinetic coefficients, which obey Onsager’s reciprocity relations [20,21]. The heat flow equation considering the endothermic processes and the exothermic Peltier effect is given by

(1) $$\nabla \cdot \left(\kappa \nabla T\right)-TJ\cdot \nabla \alpha =-J^{2}\rho ,$$

where κ, J, α and ρ are the thermal conductivity, current density, Seebeck coefficient, and electrical resistivity, respectively. It should be noted that κ(T), ρ(T) and α(T) are dependent on the temperature. Considering the Seebeck effect, the equation of the electric field in is given by

(2) $$\nabla \cdot \left(\sigma \nabla V+\alpha \sigma \nabla T\right)=0.$$

The electrical conductivity is defined as σ=1/ρ and *V* is the electric potential. The Peltier effect, which explains the heat flux **q** in the thermoelements, is expressed by

(3) q=κ∇T+αJT.

The differential Equations (1)–(3) must be solved simultaneously in a three-dimensional space: We can obtain the solution by solving these simultaneous partial differential equations with the finite element method. By obtaining the solution of the above equations, the heat transfer rate of the cold side ($Q_{c}$) and the heat transfer rate of the hot side ($Q_{h}$) of the TEG can be obtained.

The output power (P) of system can be expressed as the difference between $Q_{h}$ and $Q_{c}$ or in terms of load resistance $R_{L}$

(4) $$P=Q_{h}-Q_{c}=I^{2}R_{L}.$$

The voltage is defined as $V=\alpha \left(T_{h}-T_{c}\right)$ and the electric current is expressed as

(5) $$I=\frac{V}{R_{L}+R_{int}}=\frac{\alpha \left(T_{h}-T_{c}\right)}{R_{L}+R_{int}},$$

where $R_{int}$ is the internal resistance of the TEG. The efficiency of the thermoelement η is given by

(6) $$\eta =\frac{P}{Q_{h}}=1-\frac{Q_{c}}{Q_{h}}.$$

**Figure 1:**
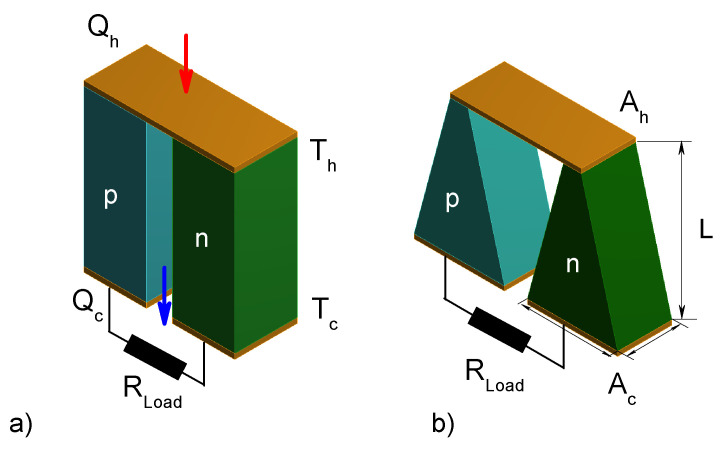
Schematic diagram of two geometrical shapes of micro thermoelectric generators (μTEG) (**a**) rectangular and (**b**) trapezoidal.

The μTEG model is composed of a p-type and one n-type thermocouple, three copper electrodes, and load resistance, which are electrically connected in series and thermally in parallel as shown in Figure 1.

It has been reported the fabrication of thermoelectric microdevices with thin thermoelements, improve the power density and supply enough energy to power a Seiko wristwatch [22,23]. So in this work, the dimensions reported by Kishi et al. [23] were used. The μTEG with rectangular shape considered in our study consists of two thermoelements with dimensions 120μm ×120μm ×300μm, connected by a copper plate 290μm ×120μm ×10μm.

Oki et al. [12] showed that the geometric modification in thermoelements improves the performance of a thermoelectric device with a slight inclination. Therefore, in this work, we performed the analysis of a trapezoidal shape where the angle was set at $\theta =72^{\circ }$, to determine the influence of the geometric parameters and $R_{L}$ on the performance of μTEG. The dimensions of the thermoelectric elements for the trapezoidal prism are 300μm in length, 40μm in width for lower side, 200μm for upper side and 120μm of thickness.

The parameter with the most influence in the selection of materials is the figure of merit. Therefore, in this work, we consider the thermoelectric properties of two PbTe nanostructured materials with doping of SrTe and Na, recently proposed by Biswas et al. [19]. The materials present with characteristics such as: Decrease in thermal conductivity with the increase in temperature, increase in the figure of merit and power factor with the increase in temperature. From its experimental data, a polynomial adjustment was proposed for the thermoelectric properties. These properties are shown in the Table 1.

Where $\alpha_{p}=-\alpha_{n}$, $\kappa_{p}=\kappa_{n}$ and $\rho_{p}=\rho_{n}$. The materials shown in Table 1, work with a temperature on the cold side of $T_{c}=300$ K and with the hot side temperature $T_{h}$ in the range from 310 K to 860 K.

**Figure 2:**
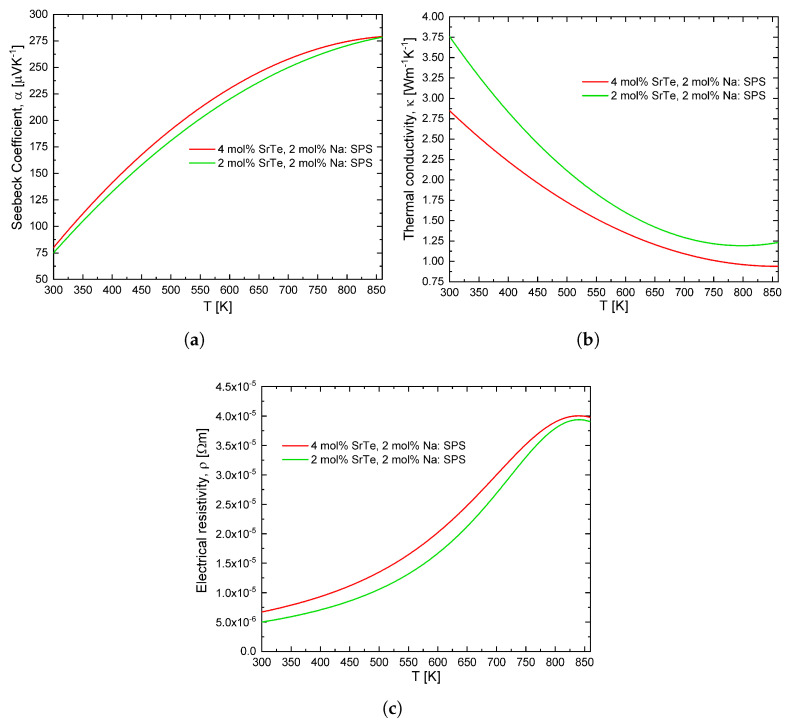
Polynomial approximation of experimental data: (**a**) Seebeck coefficient dependent on temperature, α(T), (**b**) thermal conductivity dependent on temperature, κ(T) and (**c**) electrical resistivity dependent on temperature, ρ(T) (example data for a PbTe–SrTe 4 mol% doped with 2 mol% Na and PbTe–SrTe 2 mol% doped with 2 mol% Na sample) [19].

Figure 2 shows the polynomial adjustment proposed in this work, it is a consistent approximation to the experimental behavior of the values determined by Biswas et al. [19]. The maximum value of the figure of merit used in this work is 1.8 because we consider a maximum temperature of 860 K. In general, for similar doped alloys, one has $\left|\alpha_{p}\right|\approx \left|\alpha_{n}\right|$, $\kappa_{p}\approx \kappa_{n}$ and $\rho_{p}\approx \rho_{n}$ approximately. Therefore, we use the same material for both thermoelements with these conditions.

## 3. Results and Discussion

**Figure 3:**
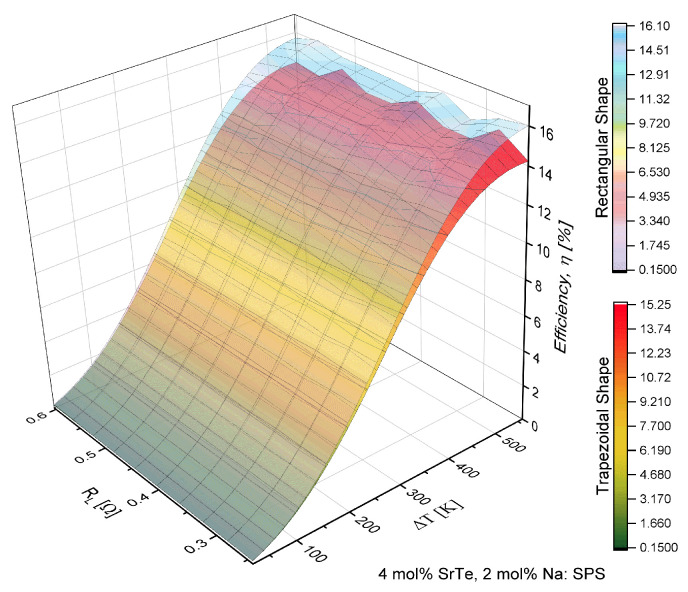
Efficiency (η) in function of load resistance $\left(R_{L}\right)$ and temperature difference (ΔT) for a μTEG with rectangular and trapezoidal thermoelements for 4 mol% SrTe.

**Figure 4:**
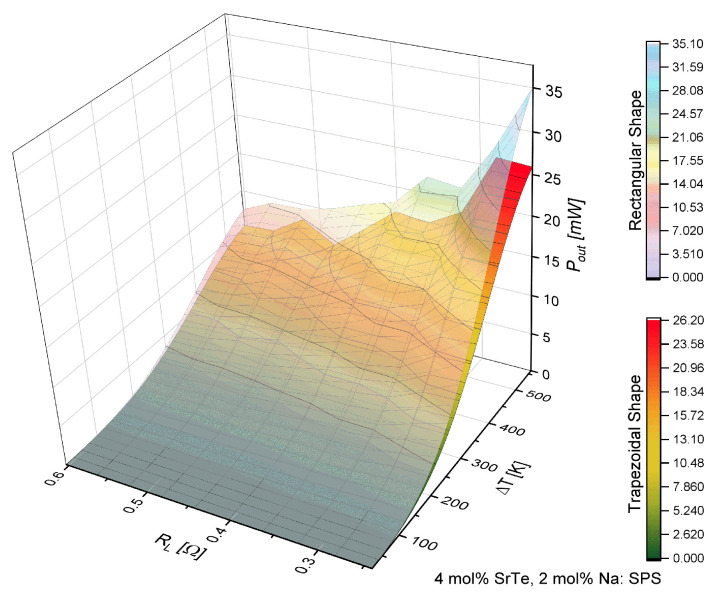
Output power $\left(P_{out}\right)$ in function of load resistance $\left(R_{L}\right)$ and temperature difference (ΔT) for a μTEG with rectangular and trapezoidal thermoelements for 4 mol% SrTe.

The results showed in Figure 3 and Figure 4 were determined for the material with doping of 4 mol% SrTe. Figure 3 shows the increase in efficiency (η) with the increase in the temperature difference (ΔT). However, there were inflections in the efficiency behavior with the increase in the load resistance $R_{L}$. This behavior was more noticeable with the change of the geometric shape, i.e., the inflections in the efficiency were notoriously prominent in the trapezoidal prism, showing an increase and then a decrease in the values of efficiency.

Figure 4 shows that the power increased with the increase of the temperature difference but decreased with the increase of load resistance. This fact is consistent with the behavior reported by Kinsella et al. [6] and Parveen [8]. However, inflections were observed in the response of output power similar to efficiency.

**Figure 5:**
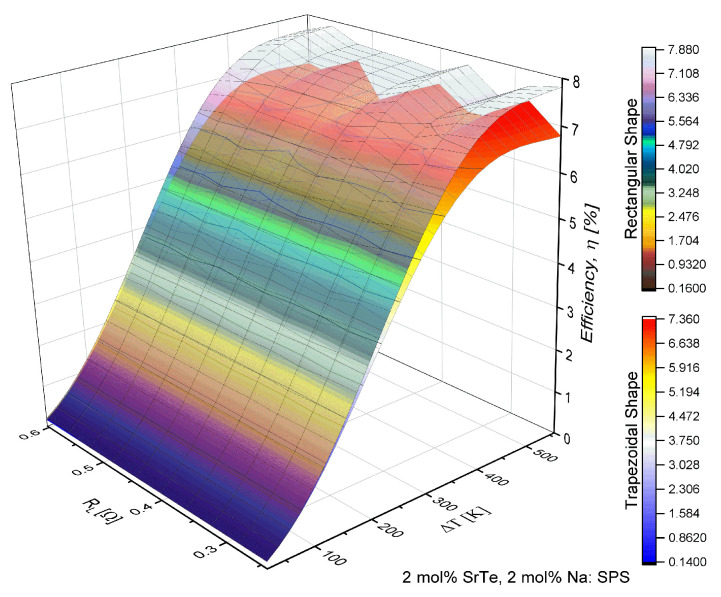
Efficiency (η) in function of load resistance $\left(R_{L}\right)$ and temperature difference (ΔT) for a μTEG with rectangular and trapezoidal thermoelements for 2 mol% SrTe.

**Figure 6:**
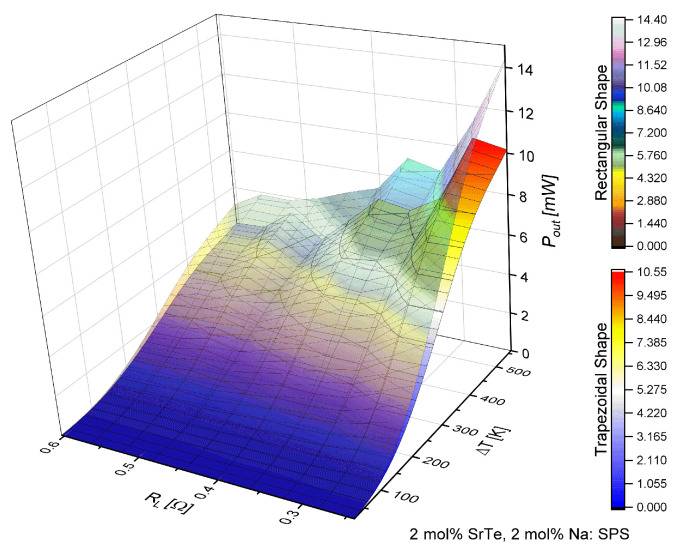
Output power $\left(P_{out}\right)$ in function of load resistance $\left(R_{L}\right)$ and temperature difference (ΔT) for a μTEG with rectangular and trapezoidal thermoelements for 2 mol% SrTe.

Figure 5 and Figure 6 show the results obtained for a material with a doping percentage of 2 mol% SrTe. Figure 5 shows that efficiency increased with the increase in temperature with a similar behavior shown in Figure 3 observed. The efficiency was variable with the increase in load resistance. More precisely, the efficiency showed peaks or maximum values in a determined range of $R_{L}$ and then decreased to minimum values. This behavior was remarkable when the temperature was higher than 600 K. The fact is attributed to the properties of the material consistent with the behavior of the power factor reported by Biswas et al. [19], which shows certain oscillations that affect directly the values of efficiency.

On the other hand, Figure 6 shows the behavior of the output power in a similar way as Figure 4; the power increase was observed with the increment of ΔT, while with the increase in load resistance decreased. However, a significant decrease of $\left(P_{out}\right)$ is shown, and the inflections shown with an increase in power in values of $R_{L}$ were similar.

Figure 3, Figure 4, Figure 5 and Figure 6 show the objective of this work, namely the behavior of efficiency and power for different values of load resistance at a given temperature difference. They show the effect of $R_{L}$ on the performance of the device. Also, our results show the maximum or minimum values of efficiency and output power at high temperatures for ΔT>300 K for different values of $R_{L}$. Notice that the performance of the device exhibited points of inflection in both efficiency and out power. Our work expanded the study on the application of nanostructured materials, to improve the performance in thermoelectric devices and to determine which are the optimal operating conditions of the proposed μTEG, mainly when the load resistance influences the performance of the device. It is a non-trivial condition for its operation.

Table 2 shows the maximum values obtained for the efficiency and output power of μTEG, these values were obtained with ΔT=560 K.

According to the values shown in Table 2, for the material of 4 mol% it was demonstrated that the rectangular shape had the maximum efficiency, while with the trapezoidal shape it decreased 0.84% compared with the rectangular shape. It should be noted that the maximum efficiency of the rectangular prism was obtained with $R_{L}=0.56\;\Omega$ while for the trapezoidal shape it was accomplished with $R_{L}=0.28\;\Omega$.

The maximum output power was obtained with the rectangular shape and decreased 8.8 mW with the trapezoidal shape. The maximum output power was obtained with $R_{L}=0.24\;\Omega$ for the rectangular shape and $R_{L}=0.28\;\Omega$ for the trapezoidal shape. These results show the effect of the geometric shape of the thermoelements in the μTEG on the maximum efficiency and power with different values of load resistance.

Comparing the same geometry of the thermoelements but with a different material concentration of 4 and 2 mol% SrTe, it was observed that the efficiency decreased as shown with the power factor, that is, decreased with high-temperature ranges; this behavior was oscillating. The effect of the change of material showed a decrease in efficiency for the rectangular shape of 51%, while the power decreased 59% respectively. For the trapezoidal case, the efficiency dropped by 52% and output power decreased by 60%, being a similar decrease between different materials.

**Figure 7:**
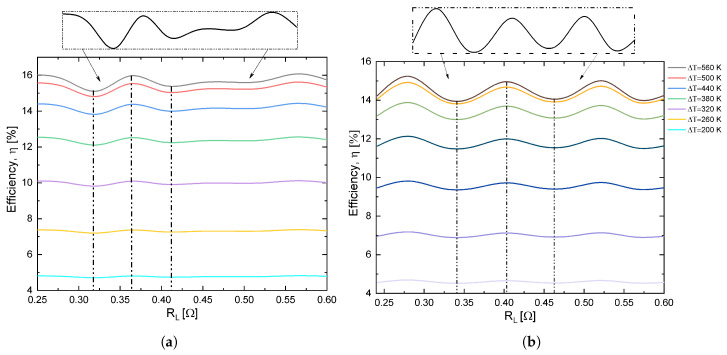
Inflections in efficiency with (**a**) rectangular and (**b**) trapezoidal thermoelements in function of load resistance $\left(R_{L}\right)$ at different temperatures for 4 mol% SrTe.

Figure 7a,b shows that the inflections in the efficiency with respect to the load resistance increased or became more acute for a high-temperature range (560 K), while when the temperature decreased the peaks were of lower intensity. The rate of change in efficiency with the load resistance varied for different intervals when we increased load resistance. The maximums in the inflections decreased as the load resistance increased. Note that, in the range of 0.32 Ω to 0.55 Ω the profile of the efficiency with respect to $R_{L}$ was analogous to that of Lamba et al. [11]. For non-nanostructured semiconductor material an important point is that thermoelectric output is not independent from external load. In other words, the thermoelectric is a dependent source when its open circuit voltage is constant but its output voltage is strongly dependent on external load resistance [7].

**Figure 8:**
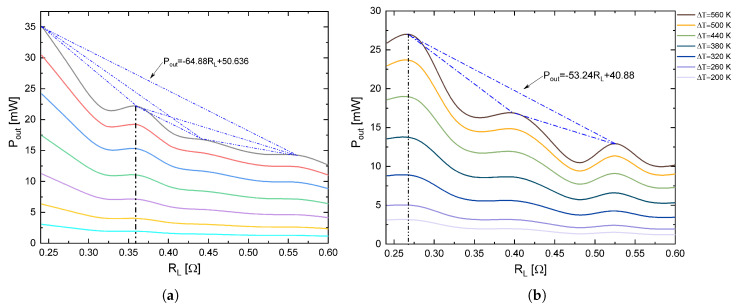
Inflections in output power with (**a**) rectangular and (**b**) trapezoidal thermoelements in function of load resistance $\left(R_{L}\right)$ at different temperatures for 4 mol% SrTe.

Figure 8a,b shows how the output power was consistent with the behavior of the power factor reported by Biswas et al. [3], that is, the power factor was modulated with a high-temperature value. In the temperature range from 500 K to 860 K, these inflections were manifested in power and efficiency when the load resistance changed. An important point to note is the fact that the modulations in efficiency and power were shown more when the geometry was not the conventional one, that is, the minimum and maximum performance values of μTEG were more noticeable when the thermoelements were trapezoidal.

Recent research has determined that the performance of nanostructured materials is optimized by controlling the dispersion of phonons with a mesoscale hierarchical structure, so phonons that carry heat with long mean free path can be dispersed. This approach resulted in a realistic perspective of part of the recovery of waste heat [3]. Our results show that the performance of μTEG shows a significant improvement compared to conventional devices, hence this result can be used for the design of TEG’s for the recovery of residual heat with high temperatures.

### Spatial Temperature Distribution

**Figure 9:**
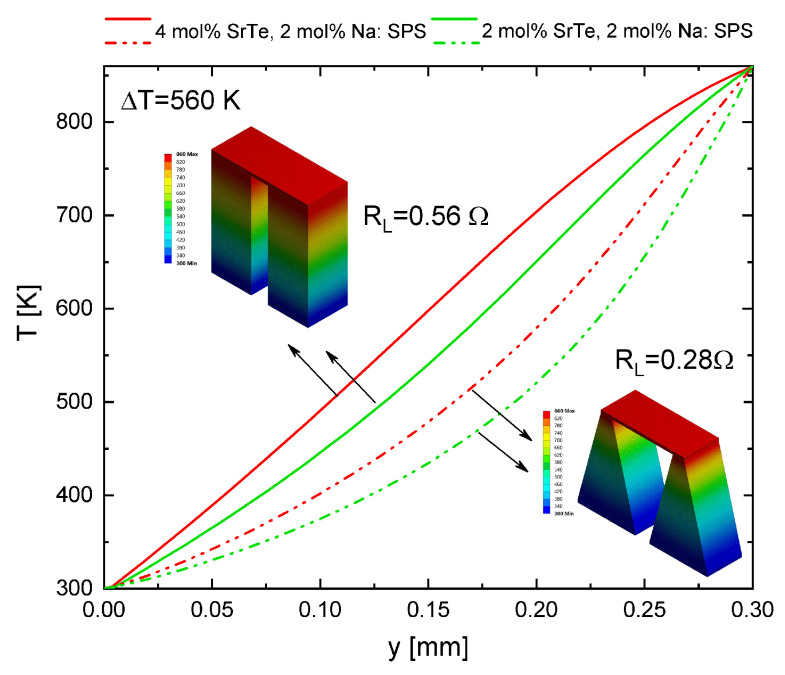
Temperature distributions of the μTEG, for rectangular and trapezoidal geometric shapes for two materials: PbTe–SrTe 4 mol% doped with 2 mol% Na (red lines) and PbTe–SrTe 2 mol% doped with 2 mol% Na (green lines) at ΔT=560 K.

Figure 9 shows the spatial temperature profile for the rectangular and trapezoidal geometric shape. The value of the load resistance was fixed for the same geometry. Two different concentrations of the material proposed in this work were used to show the behavior of the μTEG and the effect of the modification of the material in the model. Therefore, this work can be extended and generalized to other materials. The trapezoidal shape shows that, when the hot temperature was maintained in the area which has a smaller cross-section, the heat transfer was reduced because it had a reduction in the surface. Thus, the hot side was maintained at a higher temperature in the trapezoidal shape, as seen in Figure 9. Unlike the rectangular shape, the 3D temperature distribution for the trapezoidal shape was shown as a fan-shaped irregular increment while in the rectangular shape it was linear.

## 4. Conclusions

The interaction between μTEG and load resistance plays an important role in the optimization of a design, since it has been shown that its performance depends on the relationship between $R_{L}$ and its internal resistance. If the temperature dependence of the materials is considered, the resistance of the thermoelectric device changes with the increase in temperature so that the optimum relation between resistors is affected. The load and heat transport properties for nanostructured materials vary with respect to the composition. Our results show that when changing the composition of the material, the efficiency and power performance parameters were significantly affected leading to points of inflection in the performance of the μTEG. When decreasing the concentration of SrTe, the efficiency decreased around 50 percent. In comparison with bulk semiconductors, the maximum efficiency obtained was in a range of 5% to 8%, while, for the nanostructured materials studied in this paper, it was in a range of 7% to 16%. Thus, for the same geometry, the efficiency and power in non-nanostructured semiconductor materials in bulk were lower than for nanostructured materials.

An important result in this work is that nanostructured materials had maximum and minimum values (inflection points) as the load resistance changed, and that such inflections are not present in bulk non-nanostructured materials. This result shows the generalization of an important point, namely that the thermoelectric output was dependent on external load. Thus the nanostructured μTEG is a dependent source which its performance is strongly dependent to the external load resistance.

The nanostructured thermoelectric materials proposed in this paper showed a modulated behavior with respect to the load resistance, that is, the efficiency increased or decreased with the increase of the load resistance, and this effect was highlighted in ranges higher than 600 K. The results show that the maximum efficiency of μTEG was obtained with a load resistance value, but lower output power was developed. With respect to the geometric shape, the rectangular thermoelements obtained maximum efficiency and output power. However, the load resistance showed a more significant influence on the trapezoidal thermoelements. The results of this study can be an aid in choosing the design of nanostructured thermoelectric microgenerators, considering the inflections that are shown in the behavior of the performance of μTEG.

## Figures and Tables

**Table 1:** Thermoelectric properties of PbTe–SrTe 4 mol% doped p-type with 2 mol% Na: SPS and PbTe–SrTe 2 mol% doped p-type with 2 mol% Na: SPS.

Material		
	$\alpha_{p}=-1/1800T^{2}+T-170$	(V/K)
4 mol%	$\rho_{p}=1/\left(17/40T^{2}-4285/6T+325,000\right)$	(Ωm)
	$\kappa_{p}=3/490,909T^{2}-21/2000T+109/20$	(W/mK)
	$\alpha_{p}=-40/86747T^{2}+539/600T-153$	(V/K)
2 mol%	$\rho_{p}=1/\left(43/72T^{2}-6025/6T+447,500\right)$	(Ωm)
	$\kappa_{p}=5/483,871T^{2}-33/2000T+389/50$	(W/mK)

**Table 2:** Comparison of maximum values of efficiency and output power at ΔT=560 K.

	Parameter	Rectangular Shape		Trapezoidal Shape	
Material		$\eta_{\max}$ (%)	$P_{\mathrm{out}}$ (mW)	$\eta_{\max}$ (%)	$P_{\mathrm{out}}$ (mW)
4 mol%		16.071	35.093	15.233	26.199
		$R_{L}=0.56\;\Omega$	$R_{L}=0.24\;\Omega$	$R_{L}=0.28\;\Omega$	$R_{L}=0.28\;\Omega$
2 mol%		7.873	14.357	7.347	10.501
		$R_{L}=0.56\;\Omega$	$R_{L}=0.24\;\Omega$	$R_{L}=0.28\;\Omega$	$R_{L}=0.28\;\Omega$

## Abbreviation

*A*	Area (m${}^{2}$)
*I*	Electrical current (A)
J	Electrical current density (A m${}^{-2}$)
MFP	Mean free path
$P_{out}$	output power of TEG (W)
*Q*	Heat transfer rate (W)
$R_{L}$	Load resistance (Ω)
$R_{int}$	Internal resistance (Ω)
T	Temperature (K)
TEG	Thermoelectric generator
V	Voltage (V)
ZT	Figure of merit
**Greek letters**	
α	Seebeck coefficient (V K${}^{-1}$)
Δ	Difference
η	Efficiencyv (%)
κ	Thermal conductivity (W m${}^{-1}$K${}^{-1}$)
μ	Micro
ρ	Electrical resistivity
σ	Electrical conductivity [(Ωm)${}^{-1}$]
**Subscripts**	
c	cold side of TEG element
h	hot side of TEG element
max	Maximum values
n	n-type TEG element
p	p-type TEG element
